# Re-expression of MHC-I to treat CNS cancers

**DOI:** 10.18632/oncotarget.28917

**Published:** 2026-09-08

**Authors:** Arseniy E. Yuzhalin

**Affiliations:** ^1^Research Center for Translational Medicine, Sirius University of Science and Technology, Sochi, Russia

**Keywords:** brain tumor, CNS cancer, glioblastoma, brain metastasis, MHC-I

## Abstract

Immunologically “cold” malignant tumors of the central nervous system (CNS) evade immune surveillance largely through downregulation or loss of major histocompatibility complex class I (MHC-I), impairing CD8^+^ T cell-mediated elimination and contributing to poor prognosis. I discuss multiple strategies to restore MHC-I expression in brain cancers, including epigenetic modulation with histone deacetylase (HDAC) and DNA methyltransferase inhibitors, adeno-associated virus (AAV)-based gene therapy for direct human leukocyte antigen (HLA) class I or transcription factor delivery, and modulation of the tumor microenvironment to enhance antigen presentation. In addition, targeting autophagy and addressing genetic HLA losses are discussed as critical considerations. Effective MHC-I restoration may require integrated approaches that also enhance antigen processing and neoantigen presentation. Overcoming CNS-specific barriers and combining MHC-I re-expression with immunotherapy and chemoradiotherapy could unlock new therapeutic avenues against CNS malignancies.

## INTRODUCTION

Immunologically “cold” tumors are characterized by poor T cell infiltration and limited responsiveness to immunotherapies, such as immune checkpoint inhibitors [1]. A primary mechanism underlying this resistance involves the downregulation of major histocompatibility complex class I (MHC-I) molecules on cancer cells [2], which impairs antigen presentation and subsequent recognition by cytotoxic CD8^+^ T cells. To evade immune elimination, these tumors employ various strategies, including camouflage (e.g., reduced MHC-I expression to avoid detection), coercion (e.g., immunosuppressive signaling to reprogram immune cells), and cytoprotection (e.g., upregulation of anti-apoptotic pathways) [3]. A critical but clinically unresolved evasion strategy is the downregulation or loss of class I major histocompatibility complex (MHC-I) on tumor cell surfaces. Since all nucleated mammalian cells express MHC-I, it plays a central role in presenting antigens to cytotoxic CD8^+^ T cells and enabling immune surveillance.

In early carcinogenesis, tumor cells typically maintain high MHC-I expression; however, immune pressure from CD8^+^ T cells eliminates these MHC-I-high cells, leading to the emergence of MHC-I-low or negative clones that are less immunogenic and more aggressive [4]. Mechanisms of MHC-I inactivation include genetic deletions [5], epigenetic modifications such as methylation of DNA and histones [6], microRNA-mediated regulation [7], and interactions with normal cells of the tumor microenvironment (TME) [8].

Malignant brain tumors, such as glioblastoma (GBM) and brain metastases (BrMs), typically display MHC-I loss/downregulation accompanied by low immune cell infiltration. The failure of immune checkpoint blockade (ICB) trials in these cancers [9, 10] can be partially attributed to brain immune privilege, shaped by the blood-brain barrier (BBB) [11] and tumor cell-derived exosomes – microsized membrane-enclosed vesicles which induce upregulation of immunosuppressive macrophage pathways controlled by arginase-1, increased IL-10 secretion, and decreased human leukocyte antigen-DR isotope (HLA-DR) expression [12]. Low human leukocyte antigen (HLA) A/B/C expression in brain malignancies is associated with poor survival [13]. To overcome CNS tumor immunosuppression, several avenues for MHC-I re-expression should be explored.

## EPIGENETIC MODULATION TO RESTORE MHC-I LEVELS

Epigenetic drugs are an established and growing class of therapeutics, while many conventional drugs can alter the brain epigenome [14]. Histone deacetylase inhibitors (HDACi) enhanced the antitumor efficacy and persistence of chimeric antigen receptor (CAR)-T cells in mouse models of osteosarcoma [15], and promoted CD8^+^ T cell infiltration and function in mice with lung cancer [16, 17]. Some studies attribute these effects to HDACi-induced MHC-I re-expression [16], although other mechanisms have also been proposed. While HDACi have not yet been tested *in vivo* for their impact on antitumor immunity within the brain, panobinostat has been shown to reverse the Warburg effect in glioblastoma (GBM) patient-derived xenografts by promoting catabolism via β-oxidation [18]. Since Warburg effect involves cancer cells favoring aerobic glycolysis, producing lactate and acidifying the microenvironment, which suppresses T cell function, it is plausible that HDACi can improve antigen-specific immune responses [19]. Similarly, tacedinaline upregulated NF-κB pathway genes (partially responsible for HLA upregulation) and sensitized *MYC*-driven medulloblastoma cells to anti-CD47 immunotherapy [20]. While these studies did not directly assess MHC-I re-expression, they clearly showed a potential to augment T-cell responses in CNS tumor models. Notably, HDAC inhibitors including vorinostat, romidepsin, panobinostat, belinostat and others vary widely in their chemical properties and potency, with substantially different IC_50_ values across CNS tumor types [20]. Furthermore, MHC-I re-expression appears achievable only at sub-cytotoxic, low HDACi doses in certain contexts [16].

Given that MHC-I gene promoters are extensively methylated in multiple cancer types [21], DNA methyltransferase inhibition offers a feasible approach to potentiate cytotoxic T lymphocyte responses via MHC-I upregulation [22]. FDA-approved hypomethylating agents for hematologic malignancies include azacitidine, decitabine, guadecitabine, and zebularine [23]. DNA methyltransferase inhibition has not been systematically tested in the CNS, possibly due to poor BBB permeability of existing inhibitors [24]; nonetheless, isocitrate dehydrogenase 1 (IDH1)-mutated GBM tumors exhibit lower expression and higher DNA methylation of HLA-A/B/C genes [25], highlighting the clinical relevance of DNA methyltransferase inhibition in select CNS cancers. Along similar lines, a known MHC-I repressor histone-lysine methyltransferase zeste homolog 2 [26] is actively studied in both primary brain cancers [27] and BrMs, [28] where it contributes to immunosuppression via facilitating protumorigenic neutrophils and microglia remodeling.

## DIRECT MHC-I RE-EXPRESSION VIA GENE THERAPY

Adeno-associated virus (AAV)-based gene therapy has been successful in treating inherited disorders, and its application is now being extended to CNS cancers. Pre-clinical studies have employed various delivery routes, including intracerebroventricular (ICV) injection and systemic administration using BBB-crossing serotypes such as AAV9 and its engineered variants. Systemic injection with BBB-crossing AAVs is less invasive and potentially enables broader CNS transduction compared to local or ICV injections.

The feasibility of this approach is supported by several reports. In one study, a hybrid AAV/phage system expressing TNF achieved a 4 to 7-fold reduction in GBM tumor burden, depending on viral titer [29]. Similarly, viral delivery of interferon regulatory factor 8 to mouse GBM lesions enhanced antitumor adaptive immunity and reduced immunosuppression [30]. These successes suggest that direct induction of MHC-I expression is both technically viable and clinically relevant. It could be achieved by delivering the patient’s specific HLA-A/B/C genes or by transducing tumor cells with potent MHC-I-inducing transcription factors like NOD-like receptor family CARD domain containing 5 (NLRC5). Whereas the latter approach was justified *in silico* and tested *in vitro* [31], its efficacy remains to be demonstrated in *in vivo* models.

Despite this promise, significant challenges persist. These include ensuring efficient and reliable viral delivery to the tumor site, overcoming the profoundly immunosuppressive TME, and optimizing the design and expression of the therapeutic transgene.

Overall, AAV-based MHC-I induction of MHC-I expression to increase immunogenicity in cancer cells may offer a strategic approach to counteract CNS tumor immune evasion and potentiate anti-tumor immune responses in CNS cancers, particularly when combined with immune checkpoint inhibitors or other immunomodulatory treatments.

## REPOSITIONING PCSK9 INHIBITORS

Proprotein convertase subtilisin/kexin type 9 (PCSK9) is a protease that regulates blood cholesterol by promoting the degradation of low-density lipoprotein receptors in the liver. PCSK9 inhibitors are a major targeted pharmacotherapy to reduce low-density lipoprotein cholesterol in subjects with hypercholesterolemia.

In melanoma and breast cancer cell lines PCSK9 was reported to promote MHC-I degradation [32], suggesting a therapeutic potential in oncology. More recently, a clinical trial investigated if the systemically administered PCSK9 inhibitor evolocumab could increase MHC-I in patients with newly diagnosed or recurrent glioma undergoing resection [33]. A single pre-operative dose of evolocumab demonstrated measurable BBB penetrance, showing a tumor-to-blood ratio similar to other monoclonal antibodies. Importantly, the trial provided proof-of-mechanism by showing that tumors with higher evolocumab concentrations exhibited increased surface MHC-I expression and CD8+ T cell infiltration, and enhanced cytotoxic activity [33]. These findings suggest that PCSK9 inhibition can potentiate an anti-tumor immune response in glioma, yet the efficacy may be constrained by the BBB. It is clear that future trials should combine PCSK9 inhibitors with technologies like low-intensity focused ultrasound to enhance drug delivery to the tumor.

## MODULATING IMMUNE TME

This strategy broadly aims to influence the TME to promote MHC-I re-expression [34], achievable through several ways. First, interferons (IFNs) γ and α from activated T cells upregulate the antigen-presenting machinery including TAP, LMP proteasomes, and MHC-I, in tumor cells [35]. This enhances tumor visibility to immune cells, thereby linking non-specific cancer cell killing to a reinforced antigen-specific T cell response. Recent data show that high cerebrospinal fluid levels of IFN-γ correlate with improved survival of BrM patients, while targeted overexpression of IFN-γ suppresses BrM growth *in vivo* [36]. Interestingly, leptomeningeal IFN-γ supports maturation of conventional dendritic cells (DC) which recruit and promote proliferation of natural killer (NK) cells, collectively driving a robust antitumor immune response in the brain [36].

Second, enhancing antigen cross-presentation and MHC-I surface expression in TME-infiltrating DCs via immune checkpoint inhibition (e.g., SHP1 blockade) is promising [37]. This strategy appears especially promising in GBM, where tumor cells can be directly reprogrammed into DCs [38] and where autologous tumor lysate-loaded DC vaccine significantly improved overall survival (OS) in patients with both newly diagnosed and recurrent disease [39].

Third, MHC-I can be enhanced by disrupting cancer cell communication with resident brain cells such as microglia or astrocytes. While astrocytes are required for BrM growth [40] and are largely immunosuppressive [41], inhibiting their crosstalk with cancer cells can promote MHC-I surface expression and CD8^+^ T cell infiltration [8]. Particularly, cancer cells educated by astrocytes via extracellular matrix protein EMILIN-1 display increased levels of cyclin-dependent kinase 5 (Cdk5), a critical kinase whose expression almost exclusively limited to CNS [8]. This neuronal-like phenotype of tumor cells enables downregulation of surface MHC-I, whereas inhibiting Cdk5 with a specific inhibitor roscovitine resulted in restored MHC-I expression and improved immune responses [8]. Importantly, combination therapy of roscovitine with ICB substantially reduced BrM burden in mice, partly by boosting CD8^+^ T cell-mediated tumor killing [8]. Targeting CNS cancers with roscovitine is still in pre-clinical stages and requires further investigation to understand its full potential and limitations.

It should be noted that modulating the immune TME is highly context-dependent. Therapeutic outcomes will vary widely across patients with different genetic backgrounds and distinct histological and molecular CNS cancer subtypes.

## TARGETING AUTOPHAGY

Autophagy is an essential degradative and recycling pathway modulating the internalization and degradation of MHC-I molecules and contributing to alternative pathways for antigen processing and loading onto MHC-I [42]. Multiple groups reported that autophagy promotes immune evasion in pancreatic cancer by degrading MHC-I [43–45]. In melanoma, autophagy suppresses MHC-I expression, but can act as a positive regulator in the presence of IFN-γ [46].

Within the CNS, GBM cells promote autophagy through extracellular matrix deposition [47] and specific microRNA [48], whereas BrMs from breast cancer display higher autophagy marker expression compared to primary tumors [49]. To date, no studies have directly examined the link between autophagy and MHC-I regulation in CNS cancers. This represents a promising area for investigation, especially given that combining the autophagy inhibitor chloroquine with a STING agonist increased MHC-I surface expression and CD8^+^ T cell infiltration in tumor models [50].

## MANAGING PATIENTS WITH MHC-I LOSS

One major obstacle to re-expressing MHC-I for enhancing cancer immunotherapies is the partial or complete loss of HLA alleles in some patients. In non-small-cell lung cancer (NSCLC), up to 40% of cases exhibit MHC-I loss of heterozygosity (LOH), particularly among patients with high tumor mutational burden and PD-L1 positivity [51]. Importantly, MHC-I LOH was twice as frequent in BrMs compared to matched primary NSCLC tumors (43% vs. 27%), underscoring immunosuppression as a key feature of CNS malignancies [51]. TCGA data indicate the expression loss of one or more *HLA-A*, *HLA-B*, or *HLA-C* genes occurs in 16% of lower-grade gliomas and 12% of GBMs [52]. Furthermore, investigation of 60 GBM cases demonstrated *HLA-A/B/C* LOH in 41.4% patients [13]. Besides HLA genes, BrM patients may harbor loss of the major MHC-I component beta-2 microglobulin (β2m) [53].

Clearly, individuals with genetic deletion of HLA or β2m genes will be less responsive to therapies aimed at stimulating MHC-I expression. Direct MHC-I re-expression via gene therapy could offer a viable approach for these cases; however, no AAV gene therapy is currently FDA approved or widespread for cancer treatment. Considering poor survival of cancer patients with MHC-I loss [13], effective treatment remains challenging.

## MHC-I ACTIVATION VERSUS NK CELL ACTIVATION

It is generally believed that MHC-I downregulation/loss in cancer cells removes HLA-mediated inhibitory signals, leading to natural killer (NK) cell activation and increased cytotoxicity in the TME [54]. Indeed, human uveal melanoma cell lines with over 75% MHC-I positivity exhibited significantly reduced NK cell-mediated lysis [55]. Conversely, increased MHC-I expression enhances targeting of cancer-initiating cells by cytotoxic lymphocytes but reduces NK cell–mediated killing [56]. However, the relationship between MHC-I levels and NK cell function is complex and influenced by the tumor context, other activating or inhibitory signals, and the broader immune response.

For example, a recent study found that NK cells are not indispensable for the efficacy of an ICB regimen against MHC-I-negative tumors [57]. Additionally, therapies like anti-PD1 in melanoma patients have concurrently increased both CD8+ T cell and activated NK cell frequencies [58]. Furthermore, certain populations of CNS tumor cells such as CD133+ glioma stem cells demonstrate simultaneous downregulation of both MHC-I and NK cell activating ligands, suggesting coordinated immune evasion mechanisms [59]. Collectively, these findings suggest that re-expressing MHC-I in CNS cancers is unlikely to impair NK cell–mediated tumor killing *per se*, since these processes are not fully reciprocal.

## INTERSECTION WITH CAR-T AND NK CELL THERAPY

Chimeric antigen receptor (CAR)-T cell therapy is being investigated for CNS malignancies. Clinical trials targeting IL13Rα2, EGFRvIII, and HER2 have demonstrated transient regression of tumor mass in patients with recurrent GBM [60–62]. Because CARs recognize surface antigens independently of MHC-I, this strategy does not require classical antigen presentation. However, durable responses are uncommon due to heterogeneous antigen expression and the emergence of antigen-negative clones [63]. These escape populations retain susceptibility to endogenous CD8^+^ T cells if MHC-I is preserved, thus providing a rationale for combining CAR-T therapy with approaches that restore MHC-I expression. Furthermore, CAR-T cells engineered to secrete IFN-γ or interleukin-12 can upregulate MHC-I and antigen-processing machinery on neighboring tumor cells, broadening the endogenous antitumor T cell repertoire [64, 65].

NK cells offer an alternative therapeutic modality (Figure 1). CAR-NK cells retain native activating receptors such as NKG2D and DNAM-1 alongside the engineered CAR, enabling recognition of stress ligands frequently upregulated on CNS tumor cells independently of MHC-I status [66]. MHC-I re-expression could theoretically inhibit NK cell activity through killer immunoglobulin-like receptor (KIR) signaling; however, CAR-mediated activation can override this inhibition, particularly when MHC-I upregulation is achieved pharmacologically rather than by genomic restoration [67]. Allogeneic “off-the-shelf” CAR-NK products may additionally benefit from MHC-I mismatch, which can enhance tumor clearance through missing-self recognition without causing graft-versus-host disease [68]. Sequential regimens that administer MHC-I-upregulating agents after CAR-NK-mediated cytolysis could combine the tumor-debulking capacity of NK cells with sustained immune surveillance by reactivated CD8^+^ T cells. Preclinical evaluation of these combinations in orthotopic CNS models will be required to determine optimal dosing and safety.

**Figure 1:**
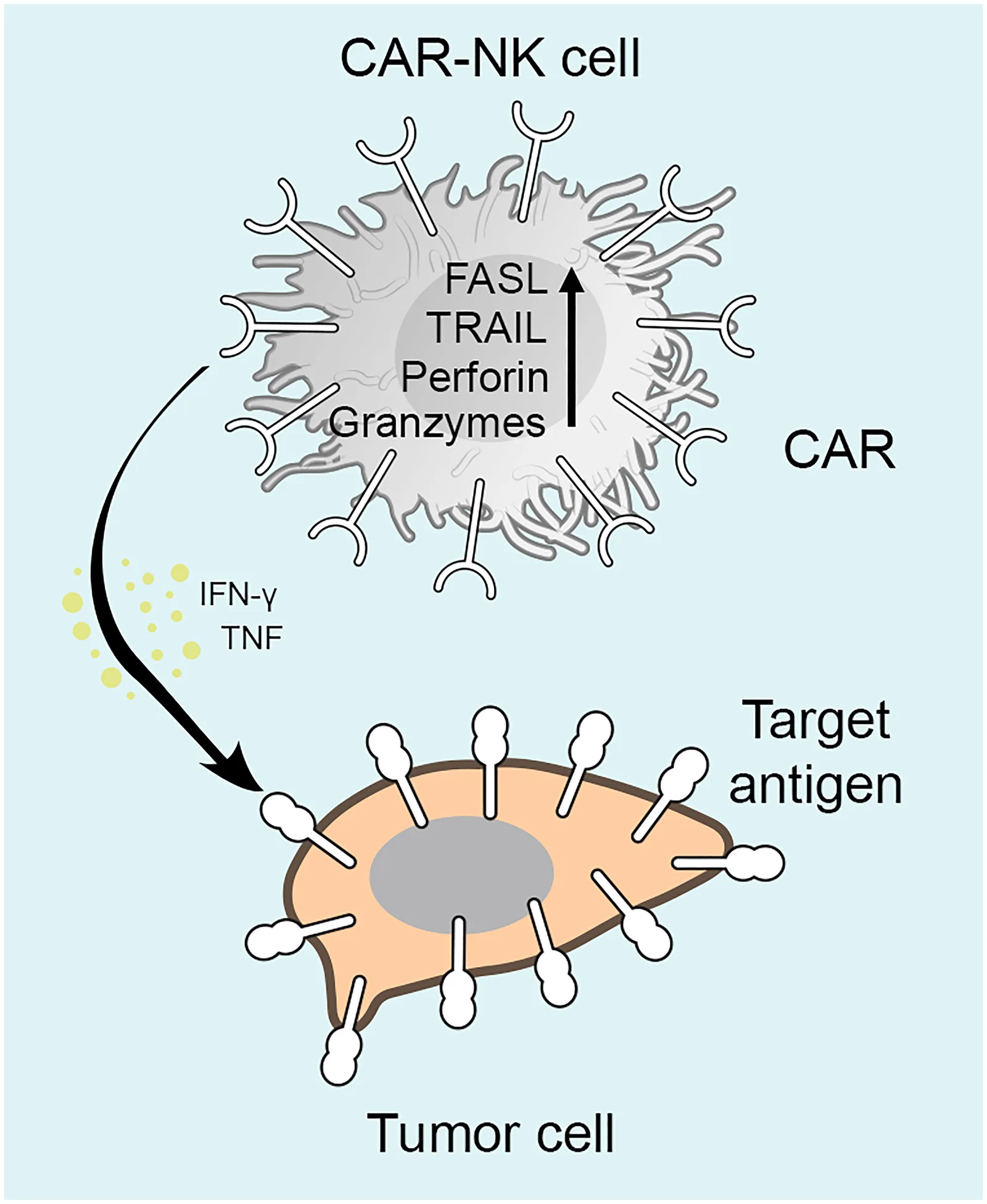
CAR-NK cell-mediated tumor cell killing. Upon engagement of the chimeric antigen receptor (CAR) with tumor-associated antigen (TAA) on the target cell, an activating immunological synapse is formed. This triggers intracellular signaling cascade within the CAR-NK cell, leading to the release of cytotoxic granules containing perforin and granzyme B into the synaptic cleft. Perforin forms membrane pores that facilitate granzyme B entry, initiating caspase-dependent apoptosis in the tumor cell. In addition, activation of death receptors through FasL/Fas and TRAIL/TRAIL-R interactions induces apoptosis in tumor cells. Additionally, CAR-NK cells retain the capacity for innate recognition through natural activating receptors (e.g., NKG2D), which detect stress-induced ligands on the tumor surface, further amplifying the cytotoxic response. This coordinated action results in tumor cell membrane disintegration and apoptotic death, while secreted IFN-γ and TNF from the activated NK cell help sustain antitumor immunity.

## MHC-I PEPTIDE REPERTOIRE AND NEOANTIGEN PRESENTATION

Restoration of MHC-I expression in CNS tumors alone may be insufficient without concurrent recovery of antigen processing and presentation pathways that shape the peptide repertoire. CNS malignancies may exhibit defects in immunoproteasome components, transporters associated with antigen processing (TAP), and peptide-loading chaperones, collectively limiting the diversity and abundance of presented peptides [69]. Given that CNS tumors generally harbor lower mutational burdens relative to other cancers [70], the availability of neoantigens for MHC-I presentation is more restricted, which can limit CD8^+^ T cell recognition and antitumor efficacy.

Furthermore, epigenetic and transcriptional downregulation of key components such as NLRC5, TAP1/2, and β2m further impair the landscape of tumor-associated peptides. The peptide repertoire is not only diminished in quantity and altered qualitatively, limiting immunogenic neoepitopes critical for effective immune surveillance [71]. Strategies to restore or enhance MHC-I antigen presentation in CNS tumors thus require integrated approaches addressing both MHC-I surface levels and intracellular antigen processing machinery.

Emerging tools like immunopeptidomics and single-cell profiling are beginning to elucidate interactions between MHC-I allelic variation, peptide diversity, and immune evasion in CNS tumors, enabling personalized neoantigen-targeting immunotherapies [72]. Combining MHC-I re-expression with neoantigen vaccination [73] or agents that promote immunoproteasome activity [74] may be necessary to fully overcome the immunosuppressive CNS TME.

## RADIOTHERAPY AND CGAS-STING PATHWAY

Radiotherapy (RT) remains a primary treatment for CNS malignancies, yet its clinical impact remains modest. In GBM, the Stupp protocol (60 Gy involved-field radiotherapy with concurrent and adjuvant temozolomide) yields a median OS of only 14.6 months compared with 12.1 months for RT alone [75]. Subsequent real-world series have reported median OS values around 15–16 months in selected cohorts [76]. Patients with BrMs show similar outcomes, with median OS following whole-brain RT is approximately 4.4 months [77], even though stereotactic radiosurgery-treated patients often have substantially longer survival in selected series [78].

Beyond its direct cytotoxic effects, ionizing radiation triggers a cascade with immunogenic potential. Initially, DNA damage generates micronuclei and releases cytosolic double-stranded DNA, which is sensed by cyclic GMP-AMP synthase (cGAS) [79]. Then, activated cGAS produces the cyclic dinucleotide 2′3′-cGAMP, which engages stimulator of interferon genes (STING) on the endoplasmic reticulum [79]. After that, STING signaling through factors TBK1 and IRF3 drives type I interferon (IFN-I) transcription, leading to upregulation of MHC-I and antigen-processing machinery on surviving tumor cells and tumor-resident dendritic cells [79]. This pathway thus links radiation-induced DNA damage to enhanced tumor visibility to CD8^+^ T cells.

Unfortunately, radiation-induced MHC-I upregulation alone has not translated into durable antitumor immunity in CNS cancers. One possible explanation is that this immunostimulatory circuit is transient and counterbalanced by immunosuppressive adaptations. Repeated RT may induce persistent IFN-I signaling, which in some preclinical settings has been associated with radioresistance and altered tumor progression [80]. In addition, RT also upregulates YTHDF2 in dendritic cells, impairing MHC-I cross-presentation and CD8^+^ T cell activation [81]. Furthermore, GBM cells can silence STING expression through promoter methylation, uncoupling DNA damage sensing from IFN-I production in the malignant compartment [82]. Consequently, the MHC-I-upregulating effects of radiotherapy are often blunted.

New treatment strategies that sustain/amplify the cGAS-STING axis following radiotherapy are needed in the clinic. Pharmacological STING agonists have shown preclinical activity in glioma models and are being explored in combination with radiation [83]. Other possible avenues include epigenetic de-repression of STING in cancer cells and optimized radiation fractionation schedules to maximize cytosolic DNA accumulation.

## FUTURE DIRECTIONS

Epigenetic, transcriptional and genetic inactivation of MHC-I are commonly exploited by CNS tumors to evade immune surveillance and drive disease progression (Figure 2) While technically possible and clinically meaningful to restore MHC-I expression in brain cancers, preclinical and clinical trials remain sparse, mostly due to unique features of the brain such as “immune privilege”. Therapeutic strategies often struggle to demonstrate efficacy due to: (i) inability to cross the BBB, collateral damage to sensitive healthy brain tissues, limited tumor infiltration, and high tumor heterogeneity eventually leading to acquired resistance (challenges primarily related to pharmacotherapy), and (ii) lack of efficient gene delivery, target specificity and risks of excessive neuroiflammation (challenges inherent to gene therapy approaches). Addressing these challenges [84] is beyond the scope of this review.

**Figure 2:**
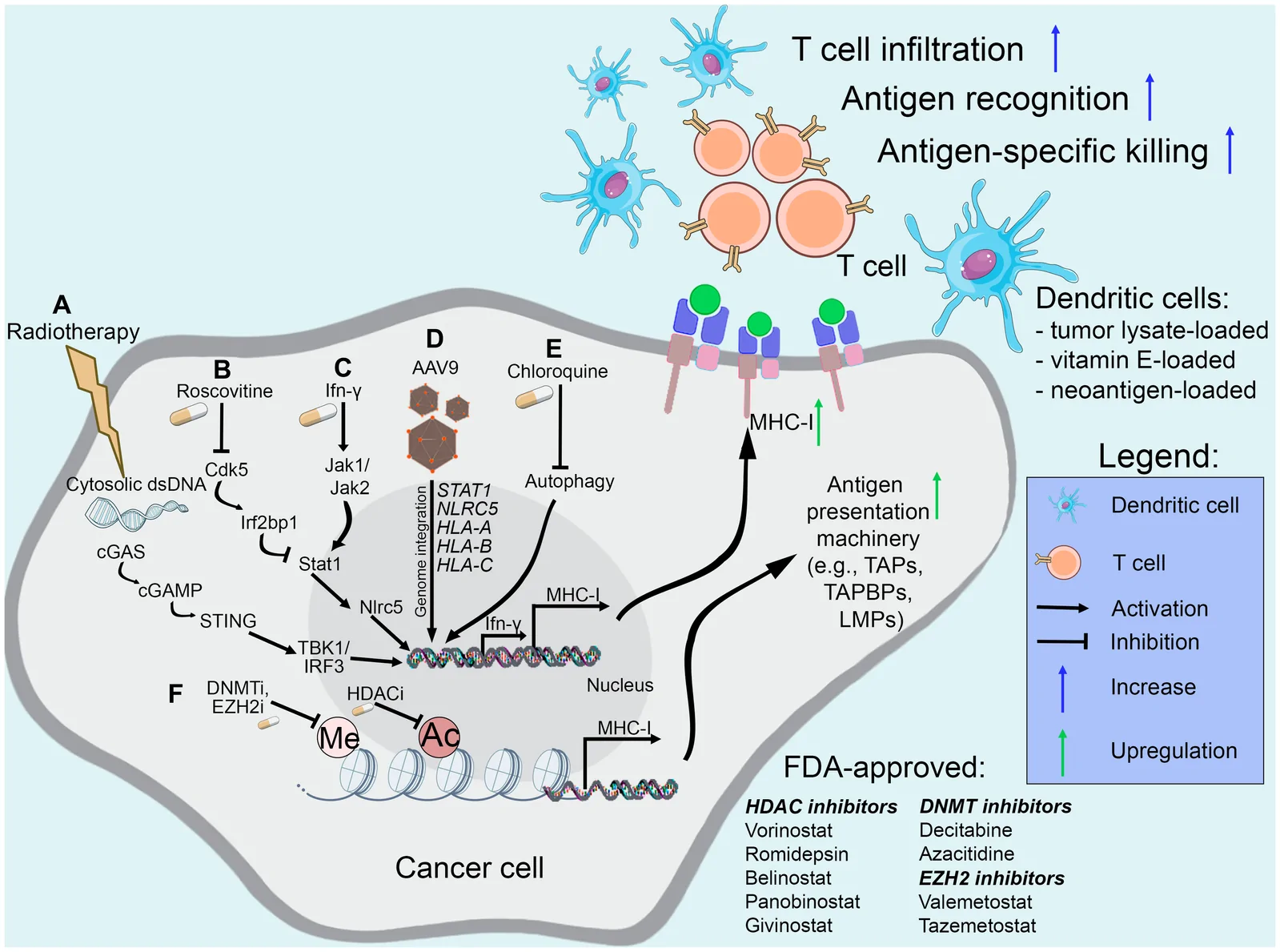
Therapeutic strategies for MHC-I re-expression in CNS cancers. Multiple molecular and cellular pathways are involved in the regulation of MHC-I expression in cancer cells of the CNS. Tumor cells in CNS cancers often exhibit diminished MHC-I surface expression, leading to immune evasion by impairing antigen presentation to cytotoxic CD8^+^ T cells. (**A**) Ionizing radiation induces DNA double-strand breaks, leading to the accumulation of cytosolic double-stranded DNA. Cytosolic dsDNA is sensed by cGAS, which catalyzes the synthesis of the second messenger cGAMP, which binds to and activates STING at the endoplasmic reticulum, prompting its translocation and subsequent recruitment of TBK1. TBK1 phosphorylates IRF3, enabling its dimerization and nuclear translocation. In the nucleus, IRF3 drives the transcription of type I interferons, which ultimately induce MHC-I. (**B**) Through phosphorylation of Irf2bp1, Cdk5 restricts phosphorylation and expression of Stat1 and Nlrc5, leading to reduced MHC-I expression on the surface of cancer cells, which eventually leads to immune evasion followed by metastatic outgrowth in the brain. Targeting Cdk5 by small molecule inhibitor roscovitine may improve CNS cancers’ management. (**C**) A pivotal inducer of MHC-I expression is IFN-γ, a cytokine released by activated immune cells or supplied externally as a potential drug. IFN-γ binds to its receptor on tumor cells, activating the JAK1/JAK2 kinases, which phosphorylate and activate STAT1. Phosphorylated STAT1 then translocates to the nucleus, promoting expression of NLRC5 and IRF-1, master regulators of MHC-I gene transcription. Additionally, p-STAT1 can also bind to GAS element to directly initiate transcription of MHC-I heavy chains. (**D**) Gene therapy using AAV vectors facilitates direct delivery of MHC-I genes or transcription factors to tumor cells, enhancing MHC-I expression. (**E**) Autophagy blockade by chloroquine inhibits degradation of MHC-I molecules, further promoting antigen presentation. (**F**) Pharmacologic agents such as HDAC inhibitors (vorinostat, romidepsin, belinostat, panobinostat, givinostat), DNMT inhibitors (decitabine, azacitidine), and EZH2 inhibitors (valemetostat, tazemetostat) are depicted suppressing epigenetic silencing, thus upregulating MHC-I genes and related antigen processing components (e.g., TAPs, TAPBPs, LMPs).

Despite the promise of restoring MHC-I expression in CNS malignancies, some critical limitations should be discussed. First, the BBB restricts delivery of most epigenetic modifiers and biologic agents. Even BBB-penetrant compounds show variable and frequently subtherapeutic concentrations within tumor-infiltrated brain regions [8]. Second, CNS tumor heterogeneity poses a challenge. MHC-I regulation is different across histological subtypes, genetic backgrounds (e.g., IDH-mutant vs. IDH-wildtype GBM), and spatially distinct regions within the same CNS tumor, potentially leading to heterogeneous therapeutic responses and acquired resistance. Third, re-expression strategies may inadvertently select for clones with complete, irreversible HLA loss (e.g., biallelic deletion or β2m mutations). Fourth, MHC-I restoration alone cannot overcome deficits in antigen processing and presentation machinery or the limited neoantigen repertoire characteristic of most primary brain tumors, which requires combinatorial approaches. Finally, the temporal window for effective MHC-I restoration relative to standard chemoradiotherapy requires optimization, as suboptimal sequencing may limit therapeutic efficacy or exacerbate toxicity. Addressing these limitations will require integrated strategies that combine MHC-I restoration with agents targeting immune evasion pathways, alongside rigorous preclinical modeling.

Undoubtedly, re-expression of MHC-I for the treatment of CNS cancers will likely achieve success only as part of multimodal regimens combining surgical resection, temozolomide and focal or whole brain radiotherapy [85]. Chemoradiotherapy acts as an immunogenic primer by inducing antigen release, creating a therapeutic window wherein MHC-I restoration and subsequent immunotherapies can be most effective. Strategically scheduling MHC-I restoration post-chemoradiotherapy could potentiate durable antitumor immune responses and improve clinical outcomes.

## Abbreviations

MHC-I: major histocompatibility complex class I
CNS: central nervous system
IFN-γ: interferon gamma
TAP: transporter associated with antigen processing
TAPBP: TAP binding protein
LMP: low molecular mass polypeptide
AAV: adeno-associated virus
